# An Address, Introductory to the Second Course of Lectures in the Atlanta Medical College

**Published:** 1856-08

**Authors:** Alexander Means

**Affiliations:** Professor of Chemistry and Pharmacy


ATLANTA
Medical and Surgical Journal.

VOL. I.] AUGUST, 1856. [NO. 12.

### ORIGINAL COMMUNICATIONS.

### ARTICLE I.

*An Address, introductory to the Second Course of Lectures in the Atlanta Medical College.* By ALEXANDER MEANS, M. D., Professor of Chemistry and Pharmacy. Published by the Class, May 1st, 1856.

*Young Gentlemen of the Medical Class:*

A new era has dawned upon the profession of Medicine in the South, and in accordance with the general progress of the age, it has, within our favored borders, widened its sphere of action, amplified its resources, and based more deeply, in established truths, its claims to the confidence of an intelligent and discerning public.

Acting under the general impulse of the age, and with the encouraging sanction of popular sentiment, to sustain their honorable purpose, this interesting body of young gentlemen from the surrounding country and from adjoining States have honored us with their presence to-day. We rejoice to welcome with them this large assemblage of intelligenc and beauty, who have vouchsafed to shed the light of their countenance upon this opening scene.

Encouraged by the same sanctions, and sprung by the promptings of that generous ambition which struggles to be equal to the existing crisis, I am authorized, as the organ of this young and promising Institution, and in the name of its Trustees and Faculty, joyfully to welcome you to her virgin Halls, and pledge you the benefit of her kindest attentions. Nay, more—we tender not merely the cold salutation of practiced lips, that coin for the occasion the phlegmatic phrases of a formal greeting, but with those bounding emotions which characterize the latitude from which we hail, we throw open our doors, and hands, and hearts, and meet you with the warmth of a southern welcome.

So far as we are aware, no similar assemblage, and under similar circumstances, meets at this season throughout the length and breadth of our sunny land. From the Atlantic coast, on the East, to where the wide wave of the Pacific washes the land of gold, in the far West—over a continental stretch of three thousand miles—and, again, from the city of marble palaces, in the cold North, over a sweep of two thousand more, to the sun-lit capitol of the Montezumas, in the South—not one solitary Medical class congregates for instruction under the auspices of a chartered College organization, whose Sessions are held, and whose Degrees conferred, only during the summer months, save that which is now welcomed to the Railroad city of Georgia.

We meet here, therefore, young gentlemen, from the four cardinal points of the compass, to prosecute, for four successive months, the studies of an ancient and honorable profession. We meet under the influence of common habits and common interests—surrounded by the same peculiar economy, and bound together by kindred ties. The whole field of medical science lies out in inviting richness before us, courting the edge of the reaper's blade, and it is ours to gather and garner, for future use, the wealth of the whitening harvest.

You have chosen a profession, gentlemen, which, for dignity and benevolence, stands second to none which has ever elicited the powers of the human intellect or stirred the emotions of the human heart, *save one*, and that is invested with nothing less than the authority of heaven, and is crowned with the honors of the skies. And yet, with the consecrated *ministry* your profession is in holy alliance, standing reverentially around the same bed-side, executing its mission of mercy to suffering humanity, and marshaling all its resources to succor the "human form divine," as it tremblingly topples upon the confines of eternity, but ever deferentially yielding to the still loftier aims of enlightened piety, which points the *nobler part* to the Cross and its atoning victim.

And yet you need not expect to win and wear your professional honors, gentlemen, unscathed by the tongue of slander or the shafts of hate. Regular scientific medicine must ever provoke the hostility of shallow pretenders and heartless empirics, who dare not wear her livery or grace her ranks. The very assemblage of her virtues—the extent of her learning, and the range of her success, but serves to shame the arrogance of ignorant aspirants, and arouse the rancor of picayune pill-mongers. But she has nothing to fear. Advancing knowledge raises new ramparts in her defence, and reposing upon her conscious worth, and the omnipotence of truth, she calmly awaits the inevitable issue, when the allied powers, which have so long hopelessly bombarded her granite battlements, disheartened and exhausted, shall withdraw their sinking myrmidons and admit her claims to the confidence of the nations. Why, then, should her enlightened friends and vindicators fear the flippant philippics of certain penny politicians, whose bellowsed sides yet heave in the bootless chase for fame, or the venomous kicks of those starveling stump-suckers, who feed upon the wind, and trot at bridle-length around the posts of their empirical dogmas, fiercely envious of the progress of science and the triumphs of mind?

Medicine, as we have intimated, claims a paternity of high antiquity and great learning, while medical schools, for the diffusion of professional knowledge, have been organized and sustained in almost every civilized country on the globe. Ancient Greece, in the days of her palmiest prosperity, cast the Ægis of her protection over the rising energies of the art, and in that classic age when the corruscations of her genius, from the shrouded mind of the blind bard of Scio, flashed upon the darkness of the surrounding nations, and the oratorical thunders of her greatest son, shook the throne of Macedon. Even then, proud Athens thought it no degradation to assemble her heroes and statesmen, and publicly encincture the brow of her immortal Hippocrates with a golden crown, for arresting, by his skill, the ravages of a fearful pestilence which had invaded the city at the opening of the Peloponnesian war. Governmental patronage was then extended to private practitioners, and the schools of Rhodes and Cnidos, Crotona & Cos., supplied the ablest medical instruction, and cultivated the finest minds of the age.

The "city of the seven hills," too, while teeming with senatorial talent, and doing homage to the genius of her Ciceros and Cæsars, forgot not to honor the distinguished *medical friends* of her Ciceros and Cæsars, and have, therefore, handed down the names of Artorius, Asclapo, and Asclepiades to challenge the respect of the 19th century.

Nor did the science and the art of medicine cease to flourish under particular masters, until the floods of vandal barbarism poured down upon the doomed city, from the plains of the North, and bore off upon their dark and turbid bosom, almost every trace of learning and refinement. In modern Europe, however, the institutions of medicine, were the first to emerge from the general inundation, and to assume rank and importance among the intellectual enterprises of the age. Oxford and Cambridge, London and Edinburg, Padua and Louvain, Tubengen and Leyden, Gottengen and Paris, have each contributed of their ample stores to the medical learning of the old world, and have trained some of the most profound minds in the lore of the profession. Since that time these institutions have been properly regarded as among the most important auxiliaries ever employed for advancing the cause of science and humanity. But the venerable ornaments of the profession who first headed the roll of distinction have long since gone to their rest, and have been followed by the bright train of Syndenhams and Harveys, Boerhaaves and Van Swietens, Hallers and De Haens, of later times, and these again within the glances of our own memory by the Rushes and Wistars, the Bartons and Chapmans, and Physics and Drakes of our own continent, who have each left their names in characters of light upon the records of the profession. And now, in this age of research and discovery, the field is full of adventurous minds, in both hemispheres, taxing their utmost powers in developing truth and enlarging the boundaries of science. Some there are, of our cotemporaries too, North and East and West and South, who boast no transatlantic origin, but who have been born and nursed under the pinions of the American Eagle, whose names are destined, at no distant day, to occupy a conspicuous place in the glowing galaxy of our professional greatness. Half a century ago, the North and the East were the main depositories of medical knowledge, and within the remembrance of your present lecturer, the Schuylkill city furnished the only organized school for public instruction to be found upon the continent. But what a change has passed over the whole moral and literary aspects of our great country! A new impulse has been given to every department of human knowledge. The public mind has been launched under a press of canvass, and sprung to its utmost tension. The discoveries of great chemical principles at the close of the last century, have been levied upon to meet the wants of a practical and driving population in the current age. Grand systems have been elaborated—stupendous plans completed as by magic, and the great interests of education, agriculture, commerce, and the arts have advanced with unexampled speed. Our time-honored profession has moved onward upon the same flood-tide. And although the very width and velocity of the current may have drifted much worthless debris along side of the noble craft, it has neither impaired the value of her cargo nor arrested the progress of her buoyant keel. She still rides gallantly on toward the port ahead, richly freighted with the scientific labors of Lavoiser and Thenard, Liebig and Davy, Brussais and Cruveilheir, Jenner and Laennec, and a thousand more, to bless generations yet unborn. In the history of the past, it is true, the pioneers of the profession beyond the Potomac, followed by the Drakes, the Dudleys, and the Caldwells of the West, who were laudibly active in elevating the standard of medical knowledge, were many years in advance of our own laggard movements. Too long, quite too long, has medical science in the South consented to lie in helpless dependence upon the bosom of the North, like a feeble foundling upon the breast of a foster mother, and to feed upon pap from the hands of strangers. But we rejoice to celebrate the day when it has, at least, passed its non-age, and has begun to assume, upon its native soil, the port and the functions of manhood.

We would not rifle a gem from the professional cabinets of the North or West, nor wither a leaf of the laurels which encircle the brow of greatness. We cheerfully render our homage and admiration to worth and genius, whether maturing amid the frosts and snows of the North, or ripening amongst the plains and rivers of the West; but vilely to apotheosize talent, or stand in crouching and servile submission to its claims, becomes not the decendants of such revolutionary sires as the Waltons and Habershams, the Sumters and Marions, the Henrys and WASHINGTONS of the South. The Southern soldier once stood shoulder to shoulder with his Northern confrier amid the smoke of battle—joined with him in the ringing shout of victory, or sunk with him face to face upon the same bed of glory; a noble rivalry of sovereign equals for their country's smiles, or their country's tears. Let the South, then, confidently enter the bloodless field before her and maintain her ancient and honorable confraternity in letters and in science, as well as in arms. Our strongest interests and gushing sympathies greet her as she comes, and bid her "God-speed" in the execution of her generous task. Give her but the training of her own sons, and the patronage of her own purse; break the spell which has so long paralyzed her own powers, and turn back upon the thirsty bosoms of her own Institutions the educational money-tide which has flowed for half a century into the bloated coffers of Northern schools. Then convince her of her boundless resources—unseal her eyes to the vision of her future glory, and above all, let the Angel of the Covenant but keep vigil over the cradles and colleges of her sons and daughters, and soon, very soon, shall the Southern heavens glow with the lights of sanctified learning—the Pulpit and the Press, the Lyceum and the Lecture-room, the Bench and the Bar, the Forum and the Senate-house, shedding their blended radiance over an elevated and refined people; and then shall the land of the Olive, the Palmetto, and the Pine—redolent with the moral odors of its own flowers and fruitage—bloom as the garden of God.

But while indulging in these grateful reflections, and anticipating the greatness of the coming *future*, for our profession and the world, let us not forget the *present*, nor overlook facts as we find them.

Genuine Medicine has always had its counterfeits and its antagonisms. Empiricism is sometimes seen treading closely upon her heels—simulating her port and carriage, or taking covert under her lengthening shadow. Anon she springs out into blustering hostility, makes noisy demonstrations of an immolating assault; and amid froth and fury, calling upon the world to behold her utter annihilation of her hated foe, she pitches headlong into the fight, "*pugnis et calcibus.*" Soon the dust and the shock of the encounter is over, and she recoils, crest-fallen and dishonored to behold her towering adversary, standing in conscious security, and with unclouded brow, like the monarch of European Mountains, when the vanquished storm lies howling at his feet. While the taunting ejaculation of the old Latin adage rings upon her ears from a thousand tongues, i. e., "*Montes parturiunt—nascitur ridiculus mus.*"

Some Charlatans, it must be confessed, are camp-followers to the medical corps—boast the insignia of the regular ranks, and contrive to lisp the "Shibboleth" of passport for unpracticed ears; while, by the timely promptings of Dunglison or Hoblyn, they roll out the "*sesquipedalia verba*" of the profession in startling explosions. These, however, are not the only parasitical flunkeys who have moved in the wake of power to gain the "loaves and fishes." In earlier days, scores of the same kidney followed a *greater leader.*

But under whatever phases Empiricism makes her descent upon society, mask and mysticism are indispensable to her temporary success. She closes her doors against public observation—seals the lips of her votaries, and by her bold avowals and blazing bulletins awakes the brawling plaudits of a thousand dupes. Dispel but the air of mystery which overhangs her incantations—reveal the agents which she employs, and her charm is gone. The public mind is disenchanted, and scientific medicine is again restored to popular confidence. Indeed, when Empiricism has occasionally affected cures, and attracted the attention of the intelligent and observant, it has been by wielding Jupiter's stolen thunder. The archives of the profession have been plundered of some of their cardinal truths, and the Dispensatory robbed of its legitimate supplies, to be vamped up under new forms and captivating costume, as the brightest gems of moral principle, that sparkle upon the dark back-ground of the infidel's code of ethics, have been purloined from the brilliant coronet of revelation. But let us postpone, for future notice, these blurs upon the escutcheon of the profession. It is the business of this School and of all kindred Institutions to furnish ample facilities for a thorough medical education—to insist upon honorable acquirements, and thus maintain the dignity of the healing art. These desirable results cannot be obtained without a heavy outlay on the part of the College, the untiring devotion of its Faculty, and the intellectual toils of its Students. We may safely pledge the supply of the *two former*, and to you, gentlemen, we confidently turn—appealing to your own gain, and your own glory, to contribute the *third*, and make the harmonious trio complete. Your field, it is true, is large but fruitful, and promises to patient industry, a rich reward.

The wonderful organization of the human body, and the physical and psychological laws which govern it, are to constitute your chief subjects of thought and research, and you may safely adopt as your motto, therefore, that canonized sentiment,

"The proper study of mankind, *is man*."

When the great representative of our race stood in primeval innocence and perfection in the bowers of Eden, unstained by crime, and unstricken by curse; his entire organization—his whole physical, mental, and moral machinery, moved in beautiful harmony with the laws of the surrounding universe; himself, a wonderful microcosm, exhibiting the concentrated and blended loveliness of the angelic and human natures—the climax of created excellence below the sun.

Motion, it is true, always implies the application and exhaustion of a quantum of pre-existing power. But while the complicated functions and elastic movements of the human body, necessarily involved the constant expenditure of the vital forces, creative wisdom gave access to the Tree of Life, whose energizing fruit compensated the daily waste of excitement, and contracted the wear and tear of the animal economy, so that all was health, and purity and peace in that Paradise of love. No paralysis then obtunded the sensibilities of man's noble nature. His quick ear gathered in all the harmonies of primeval music, that floated upon every gale, from the warblers of the wood; and the softer, sweeter notes which fell from the lips of woman, the queen of music and the queen of men. His ravished eye ranged, undimmed, over the blue heavens—the green forest, and the flowering landscape.

No shriveled muscle—no tottering footstep—fearful foretokens of greater ills—then damped the joys of Eden.

No burning fever-tide wilted the vigor of manhood—and no neuralgic pang shook the strong powers of life. Earth was the very vestibule of Heaven; and every faculty of man's exalted nature luxuriated in the abundance and beauty of a new world, while his spotless soul lived in light, and held communion with its God. But the hour of rebellion came, and the dread doom which followed, dried up the fountains of his earthly immortality. Every rampart of physical defence was broken down, and Disease, with her hungry legions, rushed in through every avenue of access, and for nearly six thousand years, has rioted amid the living ruins of a crushed and agonized nature. A wail of woe now breaks from the world's millions. It rolls through the palace of the prince at noonday, and reverberates from the cottage of the peasant at midnight, as a thousand maladies batten, like vampyres, upon the life's blood of our race. The sinking nations cry for help, and the Angel of Mercy drops a tear over the fate of manliness and virtue, while the ungorged grave still yawns for its prey. Yet "God, in wrath," forgets not to be gracious, and the obedient Earth stands ready from her hill-tops and vallies, to greet the anxious sufferer with a rich profusion of her minerals and roots, and barks, and fruits, and flowers—her limpid streams and her medicated springs—to alleviate the pains and heal the maladies of broken humanity. But who is competent to select, prepare, and appropriate these vegetable and mineral appliances to meet the Protean forms of disease, and arrest its ravages? Who shall undertake the delicate but Herculean task of wielding successfully these potent supplies of Nature, so as to strike and exterminate the *malady*, and not the *patient?*

It is confessedly a work of high benevolence, and vast responsibility; and demands the strongest powers of mind and the amplest resources of science. When infancy lies pale and panting upon the breast of motherhood, and beauty sinks, lung-worn and bleeding upon the couch of sickness, with the rose in ashes upon her cheek. When mature manhood rolls in fevered delirium, and a family's doom, or a nation's destiny, hangs upon the physician's skill. Who, O! who, shall meet the momentous crisis? Who interfere at this perilous hour to save from the brink of fate, and restore innocence and beauty, and worth, to the bounding hearts that love them?

Shall it be the arrogant and shameless pretender, who denounces the philosophy of the schools, and loudly bleats, like his long-bearded compeer of the flock, against the medical learning of centuries, for the very sagacious reason that his *own store-rooms* have never been the depositories of any of its wealth, and whose passport to public confidence is the sheep's *head*, rather than the sheep-*skin*. *Can* it be the miserable Shylock who has graduated in a horn-book college, peddled in pills by the dime, and rummaged for roots by moonlight; and whose cold and sordid soul, is sounding the depths of his patient's *purse*, while his lying finger feels for his *pulse?* Or *must* it be yon dozed and bloated victim of his cups, who reels over the bed-side, and looks down with seared brain, and idiotish lear, upon his helpless victim, as precious life ebbs out under his palsied finger? Never, O! never! The kindly charities of life, and the intelligence of the age, rebel against the outrage, and the trembling lips of a thousand invalids cry out against the damning imposture. Sooner, far sooner employ the horny fingers of a New-Castle coal-heaver to repair the machinery of your delicate chronometer; or call Sambo from his tunnel to tune pianos with his pickaxe, than trust to empty brains, frozen consciences, and blazing eye-balls, to guide this frail craft, laden with its priceless cargo of immortality, down the rapids, and through the breakers of dangerous disease. No, no, no; moral principle and christian benevolence, a dual sovereignty, throned in a well-balanced and cultivated mind, should direct the destinies and consecrate the labors of the good physician. *Man* should be studied—patiently studied in his complicated organism—studied with a clear head, an honest heart, and a noble purpose. Thus, and only thus does the medical profession reach that dignity to which it justly aspires, and secure the affections and command the respect of an admiring and confiding world.

The self-sacrificing spirit, and moral heroism which have so often characterized the members of our time-honored fraternity, finds scarce a parallel, save in the ranks of christian martyrdom. Even in those extraordinary cases of rashness and exposure, which transcend all common rules of action, less noble incentives are generally but too palpable. Superstitious frenzy horsed and mailed the maid of Orleans to roll back the fury of the seige, and place the imperial crown upon the head of Charles the VII, at Rheims. A vaulting, masculine ambition, prompted Margaret of Anjou at the head of her furious troops, twice to rescue her pusillanimous husband, (Henry VI,) from the captivities of York and Wakefield.

Even that great exemplar for military heroes—the mighty Corsican himself—who rode upon the whirlwind of war, and challenged the lightning of its bursting battle-cloud, was sprung to the performance of his superhuman feats by the stimulus of *national glory;* while his conquering hosts that followed in his rear, fired by the clangor of trumpets, the roar of cannon, and the shout of the charge, sent down to future years those deeds of valor which have won the world's applause. Such were the scenes, and such the triumphs of Austerlitz, Marengo, and Wagram.

But how different the motives which stir the physician to his work. How vastly different the fields of action upon which his adventurous spirit is to display its energies. We pass by the watchings, fatigues and mental struggles, which he encounters in the retirement of family homes, and away from the gaze of the public eye. We dwell not upon the occasions which find him, bending in his work of noiseless mercy over the sick bed of poverty, while the loud plaudits of the excited multitude without, roll by the humble dwelling, to flatter the pride of demagogues or inflame the ambition of chieftains. We turn to more striking scenes—more fiery ordeals.

The Demon of Pestilence has descended upon a neighboring city. He breathes his fell malaria into a thousand lungs. The very air is surcharged with death. Hospitals are crowded with the dying and the dead. Thousands more, panic-stricken and in feverish haste, fly from the widening scourge. The hall, the forum, the market, the haunts of pleasure, and the house of God, are alike deserted, and stern Desolation reigns monarch of the scene. And, as if indignant Nature would signalize her power in this work of ruin, and furnish the last

"*experimentum crucis*" by which to try men's souls—a maddening storm, robed in the blackness of night, disgorges its thunders over the stunned and trembling city, and sweeps with whirlwind fury along its drenched and desolate streets—uprooting trees unroofing churches, and yelling, as it passes, the funeral dirge that echoes from a hundred hearth-stones; while *Savannah*, our own Savannah, lies blasted and crushed under its weight of woe! In this dreadful crisis, who confronts the grim skeleton with his drawn scythe and his swath of corpses? Who bares his bosom to the shock, and does the works of mercy at the risk of life? O, 'tis a scene which might provoke an angel's gaze, and stir a sigh in Heaven! Let the world behold, and admire! The *ministers of God*, and the noble *functionaries of our profession*, pouring in to the rescue even from their distant homes, stand side by side, unblanched and fearless—breathing the dark pestilence, and heedless of the raging storm. The *one*, laboring to restore health and bind up bleeding hearts on *earth*—the *other*, pointing to an endless life, where hearts never bleed, in *Heaven*. They fight and fall together, and their memories are alike embalmed in the hearts of the good. In Augusta, too, the beautiful city of our eastern border, similar scenes transpire. In Norfolk, in Portsmouth, aye, wherever the fatal epidemic rages, the Destroying Angel finds them together at his heels—healing the sick, soothing the broken hearted, and pushing their victories of love even to the gates of Death. No warrior's wreath provokes their prurient ambition; no stirring drum, nor thrilling battle-cry arouses the transient courage of an hour. Their ambition grows to greatness, and their courage to moral grandeur, by the strange incentives of noxious vapors, reeking hospitals, and dismal death-rooms, with their fearful accompaniments of corses, and coffins, and hearses. O! let a grateful people honor that moral heroism which towers and triumphs, where common courage dies.

And now, gentlemen, for a thorough preparation to meet the wants of the world and sustain the honors of your profession. Every chair in this Institution is indispensable to the formation of a systematic whole. Like the seven prismatic tints in the gorgeous rainbow, their blended beauties strike the resplendent and sustaining arch of Scientific Medicine.

*Anatomy* invites to the consideration of all the materials employed in the construction of the vital machine: its bony frame-work, muscular, cartilagenous, nervous, and cellular tissues; its circulating fluids and its normal secretions, together with the relative position and adaptation of all its parts, and the laws of mechanical philosophy concerned in the production of motion.

*Physiology* puts together the wonderful organism, starts it into harmonious play, traces all its functional phenomena, and details all its normal actions.

*Pathology*—a cognate department—marks all aberrations from healthy action, and detects all organic lesions and diseased functions.

*The Practice of Medicine*, scans with the eye of an artist, any irregularity in the working of the machinery, penetrates the labyrinth of confused symptoms, and detects diagnostics amid the unerring insignia, which "afflicted Nature hangs out as signs of disease;" then proposes the modes of repair, and applies the remedies.

*Surgery*, while she claims to control the powerful momentum of the arterial circulation by antiphlogistics, or the lancet, takes special cognizance of local and violent injuries, where skillful taxis may restore the normal condition of the parts, or the knife, the ligature, or the saw, remove an offending member, or arrest the further progress of disease.

*Materia Medica* furnishes an extensive catalogne of *remedies*, from the three kingdoms of Nature, and from almost every country and clime—describes their botanical history, their several characteristics, and their mode of use, and leaves them in the hands of the practitioner.

*Chemistry* sweeps a still wider field, and challenges more extended research. The whole domain of Nature is hers, with every *atom* of its congregated masses, and her interesting and astounding facts are, therefore, gathered from exhaustless stores. In the prosecution of her qualitative analysis, she has ascended the heights of air, and fathomed the depth of ocean. Solids, and fluids, and gasses—the animate and inanimate worlds, and the simple elements and combined forms of super and sub-terrene bodies, have alike yielded to her profound and fearless research. The human body, in the elementary constitution of every organ, and tissue, and secretion, has been the interesting theatre of her triumphs. Still her brilliant career is onward; and like yon cometary nucleus, in its eliptical flight through the star-lit heavens, flings a long train of light in its rear, to illuminate the darkness of hitherto unexplored regions in the depths of Nature, leaving their wonders to be disclosed in coming years.

*One other* important department remains only to be briefly noticed. It stands at the vestibule of life, but moves in silence and shadows—throwing its protecting wing over innocence and beauty, and withholding its unostentacious benevolence from the gaze of a prying world.

With this glance of the work before you, gentlemen, who can consent to matriculate to-day—doze over the demands of to-morrow, and dream away the hours of a medical session, pregnant with doom to the fortune of those who hear me?

No intellectual lounger, in this age, wears the laurels which the world bestows. Great achievements are the rewards of great toil. Nor can the priceless approval of a peaceful conscience be secured, without an outlay of manly effort in the line of duty.

Little minds and little men sometimes find accidental elevations, but give them time to fall, and they will scarcely fail to find a rugged descent and a lower level. Even the contemptible Poodle may sometimes find himself elevated in mid-air by the fitful toss of an ox's horn, but returns, anon, with headlong plunge, to crawl, bruised and yelping to his kennel.

Nor can the prestige of venerable schools ever constitute a substitute for talents or for toil. Brick and mortar, columns and arches, afford no compensation for the want of thought, or the want of brains. Many a simpering idiot and drivelling dotard, have been draped from the royal wardrobe—flaunted in brocade, and traipsed through palace halls. Could mere familiarity with things of greatness confer wisdom and worth, why then, verily, the owls and the bats of Palmyra and Persepolis might have astonished the world with sage disquisitions upon architecture and history.

Then, young gentlemen, if you would occupy the Alpine heights of professional learning, look down upon the fogs of the plain you have left, and take in the grand panorama of the surrounding horizon, you must consent to over-swing the chasm, and struggle up the steep, until your adventurous foot is planted upon the proud summit.

Industry and energy, guided by intelligence, open oceans, and tunnel mountains, girdle the globe with the lightning's pathway, and send that burning messenger on its mission of mind, swinging over continents and plunging under seas.

Under their resistless force, marshaled armies sink upon the field, kingdoms are conquered, and republics grow to greatness. Nay, to their powerful appeals, science unlocks her priceless treasures, and wealth her golden stores.

But, be patient, gentlemen, and await the legitimate action of established laws, under the wise direction of a superintending Providence. Greatness is neither the child of chance nor the birth of an hour.

True, place and position may be often reached by the fickle freaks of Fortune. But solid and enduring fame, which wears an evergreen upon its brow, and challenges the respect of mankind, is no ephemeral spawn of accidental origin, but like the constantly accumulating Deltas of the Nile and the Ganges, is the result of the slow, but constant accretions of every passing hour—swelling to proportions of more commanding magnitude, as years roll away. But it may be that your outset is embarrassed with difficulties. Allow us kindly to enquire. Are you poor? Then your condition has won the compassion of the skies. For Heaven has pledged the premium of its blessing and protection to those who are your friends and benefactors. "Blessed is he that considereth the poor: the Lord will deliver him in time of trouble."—Psa. 41: 1. Poverty is the goal from which greatness has often set out on its lofty career.

But do you still shrink from the task you have attempted, because your resources are few and small? Remember, your very *position* exempts you from the jealousy and hate of the more favored, and leaves you quietly to develop your powers, until, like the young eaglet on its solitary cliff, your pinions are plumed and your muscles trained for flight. The loftiest oaks grow securely upon the lowest levels, while the raking tempest desolates the hill-tops above. Bear, then, the pressure of adverse fortune for a season, and the world shall yet see your light, as the heavier friction of the rubber evolves the more brilliant spark from the electric cylinder.

If, however, our arguments fail to inspire confidence, let facts be summoned to our aid. A host of pertinent examples, trans, as well as cis-atlantic, strikingly enforce the counsels which we give.

Virgil's inimitable pastorals, and immortal Ænid, are none the less enchanting, because he traced his paternity to no higher source than the humble occupant of a baker's shop. Nor does the glory of the great Grecian orator wane under the confession that his father once stood behind a baker's stall. The prince of living English chemists was once an obscure hostler boy; and the greatest surgeon of the world, thirty years ago, wilded the sledge hammer at his father's anvil. Such was Sir Michael Faraday, and such the far-famed Velpeau. But let our American youth only glance over the last half century of their country's history, and illustrious examples rise by scores to vindicate the power of the human mind to cut its way through poverty and obscurity, to fortune and fame.

Pause, then, with me for a moment. Look back to the "old Dominion," over the hiatus of forty years, and mark that slender, homely, flaxen-haired lad, astride of a corn-sack, slowly plashing with his clumsy farm-horse, through the swamps of Hanover, to bear his grist to the neighboring mill. He wears the costume of humble life, and from the indications of his noble countenance and expressive eye, surely deeds of greatness must be in embryo in that young brain. But let darkness rest upon the scene, and two score years pass by. Then look again. The wisdom of the nation is assembled at the Capitol. Music, the music of eloquence, with patriotism for its theme, rolls through the Senate chamber. Our beloved Republic is periled to its foundations by an official collision between the authorities of the General and State governments. The great Kentuckian has taked his powers for peace. A nation hangs in breathless suspense upon the issue, and the forest of living beings that throng the halls and galleries, seem bending forward, as under the magic lute of Orpheus, to catch the brilliancy of his tones and the majesty of his thoughts. He melts in pathos; he rises in grandeur; he soars to sublimity. He imploringly appeals to the contending sovereignties; stretches out his suppliant arms to his dissentient countrymen, and lifts the accepted *Olive Branch*, when a respondent shout of joy bursts from the hearts and lips of twenty millions of freemen. It is "the millboy of the Slashes" transformed into —HENRY CLAY!

Again, near the beginning of the present century, a sturdy plow-boy is seen in an adjoining State, thoughtfully plodding along in his upturned furrow, and sweating under the heat of a summer's sun. His contracting brow and piercing eye foretoken a history yet to come. But who shall divine his destiny? Stand still till Time shall develope it. * * * * the Congress of the United States is again in session. A fierce and powerful struggle of intellect attracts the attention of the Senate. A great constitutional question is pending, and the giant of the South has on the harness for battle. Arguments fall thick and solid as hail-stones upon the ringing helmets of his adversaries. His fiery zeal for the land of his birth, seems to fuse his soul, and send it out a seething current of liquid lava, leaving only cinders in its track; while the artillery tones of his peculiar eloquence peal like the notes of destiny through the echoing chamber.

Who would recognize the—"plough-boy of Abbeville" in South Carolina's favorite son—JOHN C. CALHOUN?

Once more, and we have done. The *South* and the *West* have exemplified the correctness of our counsels. Let the *North* report.

It is mowing time in New England. A healthy, burly, busy boy is out with his rake in the freshly shorn meadow, toiling with his father and brothers, to gather in their winter's supplies—occasionally making his homely supper of roasted potatoes, when there is no meal in the house. But here close the scene, and let oblivion cover half a century. * * * * Turn again to the Capitol of the nation. A fierce spirit has been let loose from the pit, and widely diffused a fiendish fanaticism through the whole North and East. Our beloved country again throbs with the throes of a political earthquake. Dismemberment is threatened every hour; when a noble form rises in the Senate house, with the periled fortunes of his trembling country weighing upon his heart. Mark the lofty pitch of his expanded forehead, the burning penetration of his full orbed eye, and the over-awing majesty which sits throned upon his brow. He speaks—he reasons—his deep barytone voice swells into more commanding tones, while breathless silence reigns around him. Then rising with the grandeur of the scene—planting one foot upon the North and the other upon the South, and, like the Colossus at Rhodes, overstriding the chasm opening beneath him—he proclaims in deathless accents which shall pulsate forever upon the ear of his posterity, "*I know no North, no South, no East, no West,*—I AM AN AMERICAN CITIZEN." Uttered with the roar of Niagara's thunder, and rushing with the momentum of its down-pouring flood—his eloquence unsettles the crumbling embankments of Abolitionism, and confounds her reeling ranks; while the grateful and admiring nation recognizes the little "New Hampshire haymaker" in "the great expounder of the Constitution"—DANIEL WEBSTER.

Acting, then, young gentlemen, under the classical motto—"*Labor vincit omnia,*" press on to success. He, who in this progressive age, consents to feed his *stomach,* while he starves his *brain,* may carry in his cranium a squashy mass, softly suited for a young miss's pincushion, but unfit for the high functions of life, for a place in the ranks of usefulness, or the calendar of greatness. Nor will musk and bergamot, superadded to starch, and stays, and broadcloth, meet the utilitarian demands of the age. The young exquisite who primps, and perfumes, and twirls his lank mustache, by day; and whirls toddy sticks, and drives billiard balls, by night, might, perchance, make an attractive sign for a confectionary or French bazaar. But you would as wisely substitute American *polk-stalks* to sustain the Dome of St. Peters, as confide in such rickety Liliputians to uphold the honors of Medicine, or the destinies of a great nation.

You have many encouragements to incite your zeal and spring your energies. The old are retiring from the profession, wealthy, or worn out. New territory is opening and furnishing an ampler field for the adventurous and the worthy, while the aggregate of population is increasing. Nay, however humiliating the concession, civilization itself breeds disease, and the antiquated motto of Seneca, "*Multos morbos—multa fercula fecerunt,*" is realized with fearful import in this age of French cooks, raw meats, and racy ragouts.

While, then, gentlemen, Artists are improving their models, and increasing their machinery to meet the world's wants: Commercial men multiplying their steamships, and navigating every sea: and Agriculturists spreading their guano, and enriching their soils, let us, too, determine to contribute to the rising glory of the land that cradled us. Aided by the sister Colleges that surround us, and sustained by the heads, and hands, and pens, and purses of our generous and intelligent classes, let us lift a pyramid of professional learning upon the plains of the South, that shall stand like the towering pile of Cheops—sun-lit and sublime amid the waste of centuries—a land-mark to posterity and the admiration of the world!

